# A Novel Exploration of a Combination of Gambogic Acid with TiO_2_ Nanofibers: The Photodynamic Effect for HepG2 Cell Proliferation

**DOI:** 10.3390/ma7096865

**Published:** 2014-09-24

**Authors:** Jingyuan Li, Xuemei Wang, Yixiang Shao, Xiaohua Lu, Baoan Chen

**Affiliations:** 1 Laboratory Animal Center, Nantong University, Nantong 226001, China; E-Mails: lijingyuan@ntu.edu.cn (J.L.); shaoyx@ntu.edu.cn (Y.S.); 2 State Key Lab of Bioelectronics (Chien-Shiung WU Laboratory), Southeast University, Nanjing 210096, China; 3 State Key Lab of Materials-Oriented Chemical Engineering, Nanjing University of Technology, Nanjing 210009, China; E-Mail: xhlu@njut.edu.cn; 4 Department of Hematology, the affiliated Zhongda Hospital, Southeast University, Nanjing 210009, China; E-Mail: bachen@seu.edu.cn

**Keywords:** TiO_2_ nanofibers, gambogic acid, cancer cell, PDT, associated therapy

## Abstract

As a good photosensitizer, TiO_2_ nanomaterials show potential biomedical applications, such as drug carriers or enhancers in photodynamic therapy. In this contribution, novel nanocomposites through the blending of TiO_2_ nanofibers with the active compound, gambogic acid (GA), were explored, and the results showed that GA could inhibit cancer cell proliferation in a time-dependent and dose-dependent manner, inducing apoptosis and cell cycle arrest at the G0/G1 phase in HepG2 cells. It is evident that after the GA-TiO_2_ nanocomposites were cultured with the cancer cells, the cooperation effect could effectively enhance the cytotoxicity of GA for HepG2 cells. Meanwhile, if activated by UV irradiation, under the presence of GA-TiO_2_ nanocomposites, this would lead to significant apoptosis and necrosis for HepG2 cells with a photodynamic therapy (PDT) effect. Associated with the controlled drug-release from these nanocomposites, TiO_2_ nanofibers could readily cut down the drug consumption in HepG2 cells and reduce the side-effect for the normal cells and tissue, which may be further utilized in the therapeutic alliance for cancer therapy.

## 1. Introduction

As one of the typical semiconductor materials with potential photocatalytic properties, nano-TiO_2_ is widely studied and believed to be one of the most promising nanocomposites in the field of materials science, energy science and also biology and life science [1,2,3,4]. It is well known that TiO_2_ nanomaterials possess a unique photocatalytic activity, which can produce reactive oxygen species (ROS), such as hydroxyl radical, hydrogen peroxide and superoxide, in aqueous media upon UV (ultraviolet) illumination, suggesting their potential application in photodynamic therapy (PDT) for various diseases, including cancer and, particularly, for the treatment of superficial tumors (e.g., esophagus, bladder, melanoma, hepatoma, *etc*.) [5,6,7,8,9]. The commercial titania P-25 (Degussa, Berlin, Germany) was shown to have better photocatalytic activity compared to most other TiO_2_ materials [10,11,12], which was explained by the increase in charge separation efficiency resulting from interfacial electron transfer between anatase and rutile [13,14]. New composites of TiO_2_ nanofibers, including anatase/brookite and anatase/TiO_2_ (B), were explored in previous reports with the efficient charge separation and migration of the overall properties of the semiconductor material [15,16,17].

With the high specific surface area and surface blemish structure, as well as good biocompatibility, TiO_2_ nanofibers can be used as potential drug carriers for other pharmaceutical agents [18,19,20]. In this study, we have tried to introduce these biocompatible TiO_2_ nanofibers into the research of gambogic acid (GA), as the major active component of gamboge resin secreted from *Garcinia hanburyi* trees in Southeast Asia. The synergetic effect of these composites between GA and TiO_2_ nanofibers for targeting cells, like HepG2 cancer cells, has been also explored *in vitro*, which indicates that as a promising photosensitizer, the TiO_2_ nanofibers blending with GA can optimize the use of relevant anticancer agents and facilitate the potential application for therapeutic alliance in multi-mode cancer treatment. Moreover, it is observed that these GA-TiO_2_ nanocomposites had higher pH sensitivity for efficient drug release in the targeted tumor, compared with the previous reports [21,22], indicating their efficacy to further reduce the side effects of relevant drugs to the normal cells or tissues in the blood circulation.

## 2. Results and Discussion

### 2.1. Characterization of TiO_2_ Nanofibers

**Figure 1:**
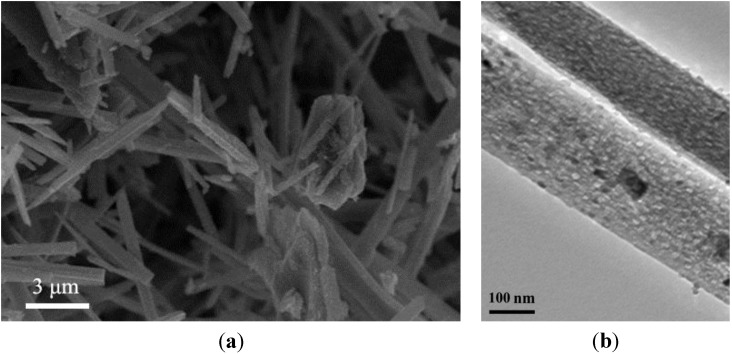
The characterization of TiO_2_ nanofibers: (**A**) SEM (scanning electron microscope) images of the TiO_2_ nanofibers; and (**B**) TEM (transmission electron microscope) image of the TiO_2_ nanofibers.

The morphology of the TiO_2_ nanofibers was prepared and characterized by Scanning Electron Microscope (SEM) and Transmission Electron Microscope (TEM). As shown in Figure 1A,B, the finer structures of TiO_2_ nanofibers were given by SEM and TEM, showing the needle-liked shape and uneven surface, with a width of about 80 nm and a length ranging from 200 to 5000 nm. The average pore size was about 5.65 nm. Anatase TiO_2_ nanomaterials have excellent activity of photosensitivity, and this kind of TiO_2_ nanofiber could have promising applications in the field of environmental, biology and medical science.

### 2.2. Loading Efficiency and Drug Release Behavior of TiO_2_ Nanofibers in vitro

**Figure 2:**
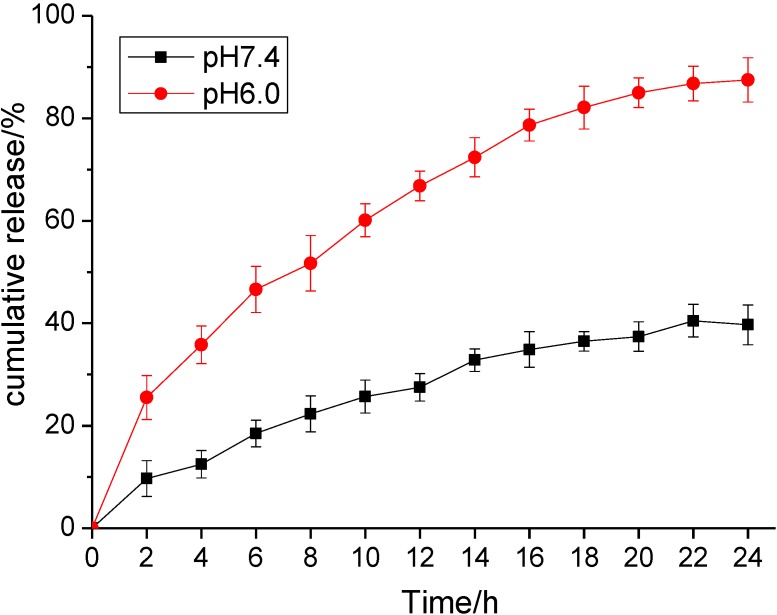
*In vitro* gambogic acid (GA) release behaviors at pH 7.4 and 6.0, respectively.

The mesoporosity and the textual porosity of these TiO_2_ nanofibers enlarge the surface-volume ratio, which provides a high specific area and exhibits the ability to absorb a larger amount of GA. According to the results, the loading efficiency and encapsulation efficiency of GA-loaded TiO_2_ nanofibers were assessed and calculated as 14.80% ± 4.5% and 62.32% ± 5.0%, respectively. The results show that TiO_2_ nanofibers may act as the anticancer drug delivery carrier. As is well known, the loaded drug should be efficiently released in the target tissue or organ, while the carried drug system should be stable in the circulation system. Here, the stimulated circumstances were carried out with the different buffer solution *in vitro*. As shown in Figure 2, the release profile of GA was dependent on the pH volume of the medium and the releasing time. The drug release at pH 7.4 was relatively slow and sustained, with a releasing ratio of about 39.7% within 24 h. However, at a lower pH, the GA release rate was much faster, with approximately 87.5% at pH 6.0 within 24 h. In pH 7.4 solution, the negative GA can be readily self-assembled onto the positive surface of these TiO_2_ nanofibers through electrostatic interaction. At a lower pH, the protonation of the drug can release the chemisorbed drug molecules into the medium and blunted the electrostatic interaction of GA and TiO_2_ nanofibers to facilitate the drug release process. This drug delivery system showed a pH-triggered release behavior, which is important in the applications of the biomedical field. It may be hypothesized that most GA might remain in the carrier for a considerable time period in the blood circulation (pH 7.4) to greatly reduce the side effects to the normal tissues. While the surrounding of the nanocomposites was changed, such as the decrease of pH value by tumor tissue or cells, a faster release of the loaded drug may occur, leading to the drug accumulation in the target sites.

### 2.3. Cytotoxicity Assay of GA and GA-TiO_2_ Nanocomposites for HepG2 Cancer Cells

**Figure 3:**
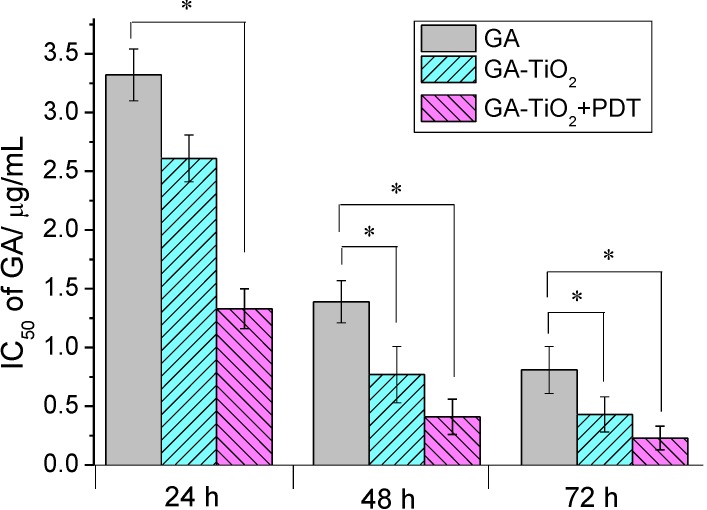
IC_50_ values of GA, the GA-TiO_2_ nanocomposites and the nanocomposites upon UV irradiation after different culture times for HepG2 cells. (* compared with the GA-treated ones, *p* < 0.05).

Gambogic acid (GA) is a natural product isolated from the gamboge resin of *Garcinia hanburyi* trees in Southeast Asia [23]. Early investigations have identified that GA was a potent apoptosis inducer, and both activation of caspases and the mitochondrial pathway are involved in GA-induced apoptosis [24,25,26]. Results from *in vitro* and *in vivo* studies indicate that GA has significant antitumor activity [27,28]. The Chinese Food and Drug Administration has approved a phase II clinical trial of GA injection as an antitumor candidate [29]. Our study indicates that GA has an apparent cytotoxicity effect for HepG2 cells, and the cell inhibition shows dose- and time-dependency, as shown in Table S1 in the Supplementary Materials. The Inhibitory concentration 50 (IC_50_) volume of GA for HepG2 cells was 3.32 µg/mL, 1.39 µg/mL, 0.81 µg/mL after HepG2 cells were treated by GA for 24 h, 48 h and 72 h, respectively (Figure 3). When TiO_2_ nanofibers were present in the drug system, the cytotoxicity of these drug-nanocomposites was further enhanced (Table S1, shown in the Supplementary Materials). It is observed that after HepG2 cells were cultured with the nanocomposites, the cytotoxicity apparently increased when compared with the treatment by GA alone, and the cell inhibition showed a dose-dependency. With the increase of the culture time, the inhibition rate remarkably increased, and the IC_50_ of GA was 2.61 µg/mL, 0.77 µg/mL, 0.43 µg/mL after 24 h, 48 h, 72 h, respectively, as shown in Figure 3. In contrast, there is little cytotoxicity for TiO_2_ nanofibers themselves under UV irradiation (shown in Supplementary Materials, Figure S1). The survival rate was more than 95% at the low concentration of less than 12.5 µg/mL. Here, there is little effect for TiO_2_ nanofibers themselves to cause the cytotoxicity of cells.

Considering the photodynamic effect of TiO_2_ nanofibers, it is noted that after being irradiated by UV light, the cytotoxicity was especially enhanced and also showed dose- and time-dependency in which the IC_50_ was 1.73 µg/mL, 0.41 µg/mL, 0.23 µg/mL after 24 h, 48 h, 72 h, respectively. Compared with the negative control, as shown in Figure 3, the IC_50_ of GA for HepG2 cells decreased by more than four-fold after being combined with TiO_2_ nanofibers and being irradiated by UV light. Moreover, compared with the HepG2 cells, the cytotoxicity of GA and GA-TiO_2_ nanocomposites for human embryonic lung fibroblast (HELF) cells was much lower than that for HepG2 cells (Table S2, shown in Supplementary Materials), indicating that these nanocomposites were much safe for normal human cells.

### 2.4. Morphological Study for the Cytotoxicity of GA and GA-TiO_2_ Nanocomposites

**Figure 4:**
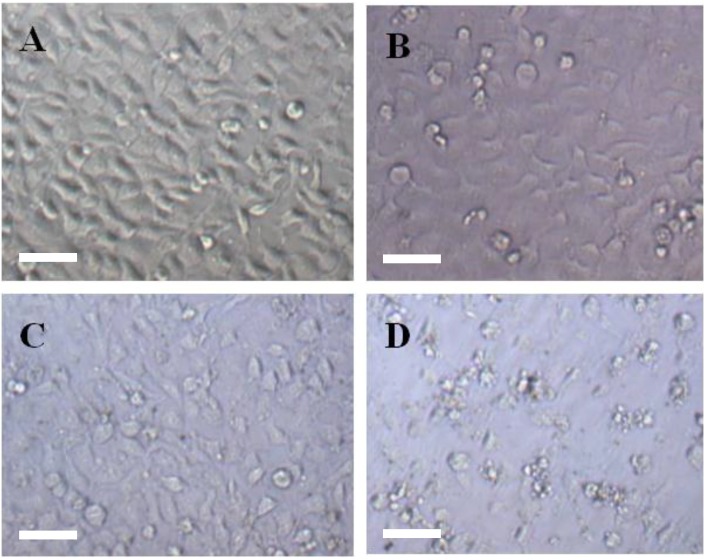
The optical microscopy images of HepG2 cells after DAPI dyeing. (**A**) HepG2 cells; (**B**) HepG2 cells treated with 0.5 µg/mL GA; (**C**) HepG2 cells treated with 5.0 µg/mL TiO_2_ nanofibers after UV irradiation; (**D**) HepG2 cells treated with GA-TiO_2_ nanocomposites after UV irradiation. Scale bar: 20 µm.

**Figure 5:**
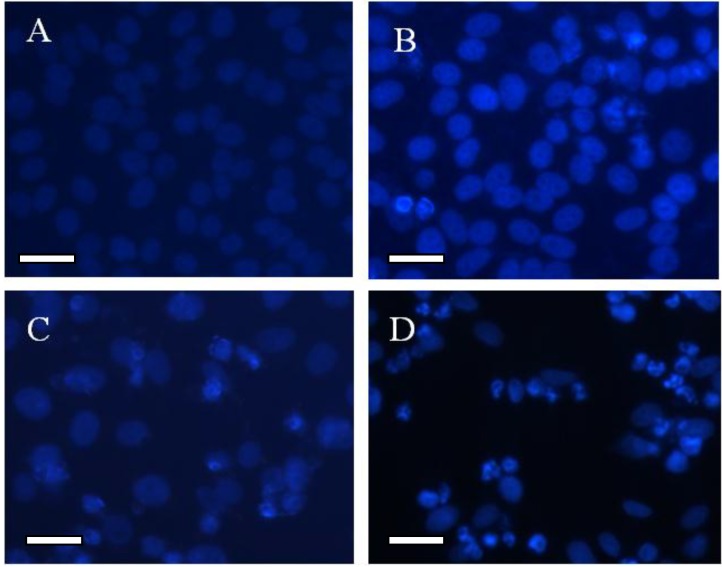
The fluorescence microscopy images of HepG2 cells after DAPI dyeing. (**A**) HepG2 cells; (**B**) HepG2 cells treated with 0.5 µg/mL GA; (**C**) HepG2 cells treated with 5.0 µg/mL TiO_2_ nanofibers after UV irradiation; (**D**) HepG2 cells treated with GA-TiO_2_ nanocomposites after UV irradiation. Scale bar: 20 µm.

The relevant morphological changes upon addition of GA and GA-TiO_2_ nanocomposites were further explored in this study. The optical microscopy characterized the changes of HepG2 cells’ morphology in the different experimental conditions. HepG2 cells in the control group displayed a normal, healthy shape, demonstrated by the clear skeletons shown in Figure 4A and Figure 5A. After treatment with 0.5 μg/mL GA for 24 h, some cells with the typical cytomorphological features of apoptosis were detected, such as cell shrinkage, chromatin condensation, margination and the presence of apoptotic bodies (Figure 4B and Figure 5B). The presence of TiO_2_ nanofibers under UV irradiation had little effect on these cancer cells, as shown in Figure 4C and Figure 5C. After being incubated with the GA-TiO_2_ nanocomposites for 24 h, the cells emitting bright fluorescence increased and displayed the typical phenomena of apoptosis, including chromatin condensation, nucleolus pyknosis and nuclear fragmentation, as shown in Figure 4D and Figure 5D.

Obviously, PDT is based on a photochemical process in which excitation energy from a photosensitizing agent is transferred to a nearby oxygen molecule, generating ROS, causing cytotoxicity [30]. These toxic species can oxidize and degrade neighboring biomolecules and subcellular structures, such as DNA and mitochondrial biomembranes, and ultimately trigger apoptosis and cell death. Compared with the organic photosensitizer, the inorganic photosensitizing agents have been widely researched in recent years [31]. Here, combined with the results of the MTT ((3-(4,5-Dimethylthiazol-2-yl)-2,5-diphenyltetra zolium bromide) assay, we tested the biocompatibility and cytotoxicity of these TiO_2_ nanofibers and the photodynamic effect after irradiation with HepG2 cells. As the control condition, the side effect of UV irradiation for the cells themselves was carefully studied in this experiment, and there is no difference compared with the cells in the absence and presence of UV under the identical experimental conditions.

### 2.5. Flow Cytometry Analysis of Relevant Cancer Cells Apoptosis

**Figure 6:**
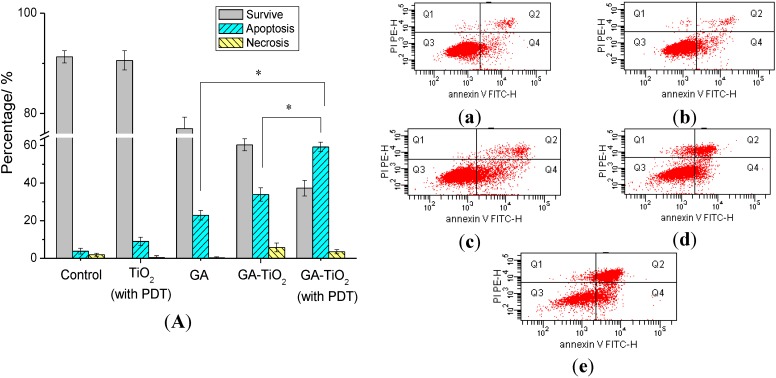
Effect of GA, TiO_2_ nanofibers and GA-TiO_2_ nanocomposite-induced apoptosis for HepG2 cells: (**A**) The apoptosis analysis of HepG2 cells; (**a**) HepG2 cells; (**b**) HepG2 cultured in TiO_2_ nanofibers with photodynamic therapy (PDT); (**c**) HepG2 cultured in 0.5 µg/mL GA; (**d**) HepG2 cultured in GA-TiO_2_ nanocomposites; and (**e**) HepG2 cultured in GA-TiO_2_ nanocomposites with PDT. (* compared with that of GA and GA-TiO_2_ without UV irradiation, *p* < 0.05).

Flow cytometry (FCM) of cell apoptosis was further investigated to estimate the genetic level changes induced by different drug systems. As shown in Figure 6, apoptosis of HepG2 cells induced by GA, TiO_2_ nanofibers or the nanocomposites was analyzed quantitatively by FCM. After 24 h of culture time, the total apoptosis ratio was 3.9% in the blank experiments of HepG2 cells (Figure 6a). In the negative system cultured with 0.5 µg/mL GA, the apoptosis of HepG2 cells was only observed to be about 22.9%. While the cells were cultured with TiO_2_ nanofibers and UV irradiation as the control system, there was an apoptosis of about 9.2% for HepG2 (Figure 6b). In comparison, with treatment by GA-TiO_2_ nanocomposites, the apoptosis rate of HepG2 cells increased to about 33.9%. Then UV irradiation was present in the above system, the rate of apoptosis cells was further increased to 59.3%, and cellular necrosis was 3.5%. There is a significant increase for the cells’ apoptosis ratio compared with the negative system and the experiments of GA-TiO_2_ cultured without UV irradiation.

From the above study, it can be observed that as the effective drug carrier, TiO_2_ nanofibers could readily enhance the cytotoxicity of GA for HepG2 cells. Compared with the negative GA, the cell survival had an obvious decrease in the presence of TiO_2_ nanocomposites. Besides, as a good photosensitizer, TiO_2_ nanofibers displayed potential capacity in PDT. After the drug-nanocomposites were irradiated under UV irradiation, the cell survival had further considerably decreased.

### 2.6. Study on the Cell Cycle Effect of GA and GA-TiO_2_ Nanocomposites

**Figure 7:**
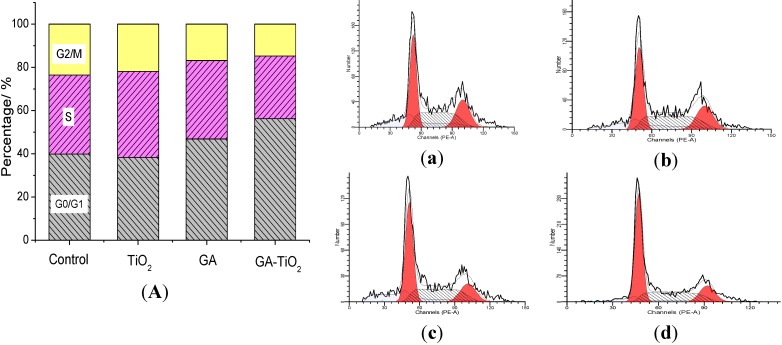
Effect of GA, TiO_2_ nanofibers and GA-TiO_2_ nanocomposites for HepG2 cells’ cycle under UV irradiation: (**A**) the ratio analysis of cell cycle phase for HepG2 cells in which: (**a**) HepG2 cells; (**b**) HepG2 cell cultured in TiO_2_ nanofibers; (**c**) HepG2 cell cultured in 0.5 µg/mL GA; and (**d**) HepG2 cell cultured in GA-TiO_2_ nanocomposites with PDT.

The effect of GA and related systems on the cell cycle was further investigated to explore the mechanism of relevant cellular interaction. As shown in Figure 7a, the ratio of G0/G1 phase was about 39.90%, and the ratio of the G2/M phase was about 23.58% in the blank systems of HepG2 cells. Results indicate that TiO_2_ nanofibers had little effect on the HepG2 cells cycle, while there was some apoptosis in the presence of TiO_2_ nanofibers under UV irradiation, as shown in Figure 7a,b. After HepG2 cells were treated with 0.5 µg/mL GA, the ratio of G0/G1 phase increased to 46.88% and the G2/M phase decreased to 16.86%, and there was no significant difference for the S phase. While in the positive system cultured with the GA-TiO_2_ nanocomposites with UV irradiation, it is observed that the rate of the G0/G1 phase especially increased to 52.26% and the G2/M phase decreased to 14.77%, as shown in Figure 7d. Thus, it was evident that the GA-TiO_2_ nanocomposites could apparently enhance the cytotoxicity of GA to inhibit the growth of HepG2 cells by perturbation of the cycle signaling network (via the G0/G1 phase).

## 3. Experimental Section

### 3.1. Drugs and Reagents

Gambogic acid (Kanion Pharmaceutical Co., Ltd., Jiangsu, China) was dissolved in dimethyl sulfoxide (DMSO; Sigma Aldrich, St. Louis, MO, USA), stored at −20 °C and then diluted as needed in Roswell Park Memorial Institute (RPMI) 1640 medium (Gibco/BRL, Carlsbad, CA, USA). MTT was purchased from Sigma Aldrich. All other regents used in this report were analytically pure.

### 3.2. Preparation and Characterization of TiO_2_ Nanofibers

The fabrication of the TiO_2_ nanomaterials (CMT-1, Nanjing University of Technology, NANJING TAIWEI technology Co., Ltd., Nanjing, China) followed the previously reported procedure in the literature [16]. Briefly, a reactant mixture with TiO_2_/K_2_O was prepared by uniformly adding K_2_CO_3_ to TiO_2_·nH_2_O and then sintered at 810 °C for 2 h. The sintered product was wet ground and dried at 60 °C, and then, 10 g of the product were soaked in 7 mL of distilled water at ambient temperature in a closed container for about 7 days, during which the potassium-rich nanophase gradually formed. When the product was totally transformed to the amorphous phase, the resultant product was suspended in 100 mL of vigorously stirred 0.1 M HCl solution until the K^+^ ions were totally removed. Then, the product was separated by filtration and washed with distilled water, followed by drying in a desiccator and at 60 °C under vacuum. Calcination of the dried Titania sample was conducted in a muffle oven at elevated temperature in air for 2 h, and then TiO_2_ nanofibers could be obtained with the tetragonal crystal structure (anatase). These TiO_2_ nanofibers were observed by TEM (JEM2100EX, JEOL, Tokyo, Japan) and SEM (JEOL JSM-5900).

### 3.3. Cell Culture

Human hepatocarcinoma (HepG2) cells and human embryonic lung fibroblast (HELF) cells (purchased from Shanghai Institute of Cells, Chinese Academy of Sciences, Shanghai, China) were maintained in dulbecco’s modified eagle medium (DMEM) (high glucose, Gibco) medium supplemented with 10% fetal calf serum (Sigma Aldrich), 100 U/mL penicillin (Sigma Aldrich) and 100 mg/mL streptomycin (Sigma Aldrich) at 37 °C with 5% CO_2_ in a 95% humidified atmosphere.

### 3.4. The Preparation of GA-TiO_2_ Nanocomposites and the Characterization of GA Loading and Release in vitro

Two milliliters of GA solution (5 mg/mL) were diluted by Phosphate Buffered Saline (PBS) (pH 7.4, 0.01 mol/L) and mixed into 2 mL TiO_2_ nanofibers suspension (5 µg/mL). This was maintained in a 4 °C freezer for more than 24 h in the dark to construct the GA-TiO_2_ nanocomposites. GA-TiO_2_ nanocomposites were separated through centrifugation at 15,000 rpm for 20 min, and the supernatant was determined by HPLC (High Performance Liquid Chromatography) with the condition of a Kromasil (Akzo Nobel N.V, Amsterdam, The Netherlands) 100-5 C18 (250 mm × 4.6 mm) column, methonal-0.1% H_3_PO_4_ (9:1) as the mobile phase, and the wave length was 360 nm.

The loading efficiency and encapsulation efficiency were calculated by the following equations:

Loading efficiency = (amount of drug in drug-loaded nanofibers/amount of drug-loaded nanofibers) × 100%.
Encapsulation efficiency = (amount of drug in drug-loaded nanofibers/initial amount of drug) × 100%.

The release studies were performed in a glass apparatus at pH 6.0 (the pH of the environment around the tumor) and pH 7.4 (the pH of physiological blood). The GA-loaded TiO_2_ was dispersed in PBS (pH 7.4, 5 mL) and transferred into the dialysis bag. The dialysis bag was then immersed in 95 mL PBS of pH 6.0 and 7.4, respectively. The release medium was continuously agitated at 37 °C with a stirrer at 100 rpm. At predetermined time intervals, 0.2 mL of the external medium was collected and replaced with the same fresh PBS. The amount of released GA in the medium was then determined by HPLC.

### 3.5. MTT Assay of GA and GA-TiO_2_ Nanocomposites in the Absence/Presence of UV Irradiation

In this process, HepG2 cells (5 × 10^3^/mL) in log phase were trypsinized and seeded in 96-well plates. After 24 h incubation, cells were rinsed in DMEM medium and incubated with different concentrations of GA or GA-TiO_2_ nanocomposites. As the positive experiments for PDT, the effect of TiO_2_ nanofibers for HepG2 cell proliferation in the presence of UV irradiation for 180 s has been investigated, in which the average intensity is 0.1 mW/cm^2^ (λ = 254 nm) at the working plane. MTT solutions were added after the treatment and incubated for another 4 h. DMSO was added to solubilize formazan crystals, and OD570 was recorded. Every experiment was repeated at least three times.

### 3.6. Cell Morphological Assessment

After being cultured in DMEM containing 0.5 µg/mL GA, 0.5 µg/mL GA conjugated with 5 µg/mL TiO_2_ nanofibers at 37 °C for 24 h, HepG2 cells were washed by PBS twice. Some were observed by optical microscopy to view the cells’ morphology; others were fixed with methanol for 15 min, stained with fluorochrome dye DAPI (Santa Cruz Biotechnologies, Dallas, TX, USA) and then observed under a fluorescence microscope (IX51; Olympus, Tokyo, Japan) with a peak excitation wave length of 340 nm.

### 3.7. Apoptosis Assay of GA and GA-TiO_2_ Nanocomposites for HepG2 Cells

HepG2 cells were treated in different concentrations of GA or nanocomposites. After being incubated with 24 h, cells were collected by centrifugation at 1000× *g* for 5 min and washed by PBS twice. Cells were harvested by trypsinization (without EDTA) and collected by centrifugation at 1000× *g* for 5 min. The pellets were resuspended with PBS and centrifuged as above, and the supernatant solutions were discarded. Subsequently, 500 µL of binding buffer were added and mixed with 5 µL of Annexin V-FITC solution (Nanjing Keygen Biotech. Co., Ltd., Nanjing, China), and then, 5 µL of propidium iodide (PI) solution were added. The resulting mixture was kept at room temperature in darkness for 10 min. Then, flow cytometry analyses were performed using Fluorescence Activated Cell Sorter (FACS) Vantage flow cytometer (Becton Dickinson, Franklin Lakes, NJ, USA), where the excitation wavelength is 488 nm and the emission wavelength is 530 nm.

### 3.8. Cell Cycle Analysis of GA and GA-TiO_2_ Nanocomposites for HepG2 Cells

HepG2 cells were treated in different concentrations of GA or nanocomposites. After being incubated for 24 h, cells were collected by centrifugation at 1000× *g* for 5 min and washed by PBS twice, then stained with PI solution. Analysis of cell cycle was performed using a Cycle Test plus DNA reagent Kit (Nanjing Keygen Biotech. Co., Ltd.). Flow cytometry analysis was performed as described previously.

### 3.9. Statistics

Data were expressed as the means ± SD (standard deviation) from at least three independent experiments. A one-tailed unpaired Student’s *t*-test was used for significance testing, and *p* < 0.05 is considered significant.

## 4. Conclusions

In summary, as photodynamic nanomaterials, TiO_2_ nanofibers were utilized in multi-mode cancer treatment. Our observations demonstrate that the GA-TiO_2_ nanocomposites could efficiently inhibit HepG2 cell proliferation in a time- and dose-dependent manner, causing the G0/G1 cell cycle arrest to induce the apoptosis of HepG2 cells. Under UV irradiation, GA-TiO_2_ nanocomposites obviously enhanced the cytotoxicity of GA molecular for HepG2 cancer cells. Being associated with the photodynamic effect for PDT, TiO_2_ nanofibers can effectively facilitate the promising clinical application of GA and other active compounds from natural products. As new photodynamic materials, many problems should be investigated before their clinical application for these TiO_2_ nanofibers. Importantly, the metabolic pathway of these inorganic nanomaterials should be carefully studied *in vivo*. Besides, the novel pharmaceutical dosage form and administration route based on these TiO_2_ nanofibers should be further explored to reduce their potential side effects. It is evident that the combination of nanomaterials, like TiO_2_ nanofibers, with the related active agents could contribute to the development of perspective anticancer candidates and promote the promising application of relevant nanostructures in clinical treatment.

## Supplementary Materials

Supplementary materials can be accessed at: http://www.mdpi.com/1996-1944/7/9/6865/s1.
