# Supplementary material for: A Novel Exploration of a Combination of Gambogic Acid with TiO2 Nanofibers: The Photodynamic Effect for HepG2 Cell Proliferation
Source: Materials (Basel). 2014 Sep 24;7(9):6865–78. doi: 10.3390/ma7096865 (PMC5456129; doi:10.3390/ma7096865)

Supplementary Materials

Table S1. The cytotoxicity of GA (gambogic acid) and relevant nanocomposites in which TiO_2 nanofibers was 5 µg/mL for HepG2 cells and the photodynamic effect of these nanocomposites after UV irradiation at different culture times.

GA (µg/mL)	Cell Inhibition/%								
	24 h			48 h			72 h		
	GA	GA-TiO_2	GA-TiO_2 (with UV)	GA	GA-TiO_2	GA-TiO_2 (with UV)	GA	GA-TiO_2	GA-TiO_2 (with UV)
0.0625	2.1 ± 2.4	4.5 ± 3.9	10.9 ± 3.7	5.0 ± 2.1	11.9 ± 2.9	21.5 ± 3.5	9.3 ± 2.4	15.9 ± 4.2	30.1 ± 3.7
0.125	4.3 ± 3.1	8.5 ± 1.6	17.0 ± 4.3	8.5 ± 3.2	25.2 ± 4.1	31.9 ± 4.4	19.5 ± 3.2	35.9 ± 3.6	53.2 ± 4.1
0.25	10.3 ± 3.2	15.1 ± 2.3	23.3 ± 4.4	14.5 ± 4.1	41.5 ± 2.9	52.1 ± 3.8	39.4 ± 3.9	47.6 ± 4.7	69.1 ± 3.5
0.50	21.5 ± 2.6	33.2 ± 3.6	49.2 ± 3.1	33.8 ± 3.2	54.9 ± 4.3	75.8 ± 3.2	48.7 ± 2.5	63.0 ± 3.7	87.3 ± 4.1
1.00	30.3 ± 3.7	46.9 ± 2.5	63.2 ± 3.5	47.8 ± 3.2	68.8 ± 3.7	87.4 ± 4.2	71.8 ± 4.1	84.5 ± 3.5	96.9 ± 4.2
2.00	35.9 ± 3.1	55.1 ± 3.16	70.1 ± 4.6	63.1 ± 2.6	86.7 ± 3.4	95.3 ± 3.9	82.1 ± 3.1	93.9 ± 3.8	96.3 ± 2.5

Table S2. Cytotoxicity of GA and GA-TiO_2 nanocomposites for HELF (human embryonic lung fibroblast) cells.

GA (µg/mL)	Cell Inhibition/%								
	24 h			48 h			72 h		
	GA	GA-TiO_2	GA-TiO_2 (with UV)	GA	GA-TiO_2	GA-TiO_2 (with UV)	GA	GA-TiO_2	GA-TiO_2 (with UV)
0.0625	0.8 ± 1.8	1.5 ± 3.8	5.8 ± 4.2	1.7 ± 2.3	3.9 ± 2.8	7.1 ± 3.0	3.8 ± 3.0	5.5 ± 2.2	10.0 ± 3.7
0.125	2.5 ± 1.5	4.3 ± 2.3	11.1 ± 4.1	3.3 ± 2.2	6.2 ± 3.1	15.2 ± 3.4	6.0 ± 1.6	10.9 ± 2.6	18.1 ± 4.6
0.25	5.6 ± 2.9	8.2 ± 3.3	16.0 ± 4.5	7.2 ± 3.1	13.1 ± 3.9	22.6 ± 3.9	10.1 ± 3.5	15.5 ± 4.3	29.8 ± 3.8
0.50	16.1 ± 3.0	20.3 ± 2.8	30.4 ± 3.2	20.9 ± 2.9	25.9 ± 4.1	35.9 ± 3.5	29.5 ± 3.9	33.9 ± 3.7	41.6 ± 4.4
1.00	30.8 ± 3.1	36.9 ± 3.6	43.2 ± 4.3	35.7 ± 3.2	40.0 ± 3.7	50.3 ± 4.6	41.2 ± 3.3	48.5 ± 3.6	56.8 ± 3.6
2.00	43.3 ± 2.7	48.4 ± 4.6	60.1 ± 4.9	57.6 ± 3.1	66.2 ± 3.0	75.3 ± 3.6	66.3 ± 2.5	73.0 ± 3.8	86.9 ± 4.5

Figure S1. The cytotoxicity of TiO_2 nanofibers at 48 h and the photodynamic effect after UV irradiation.

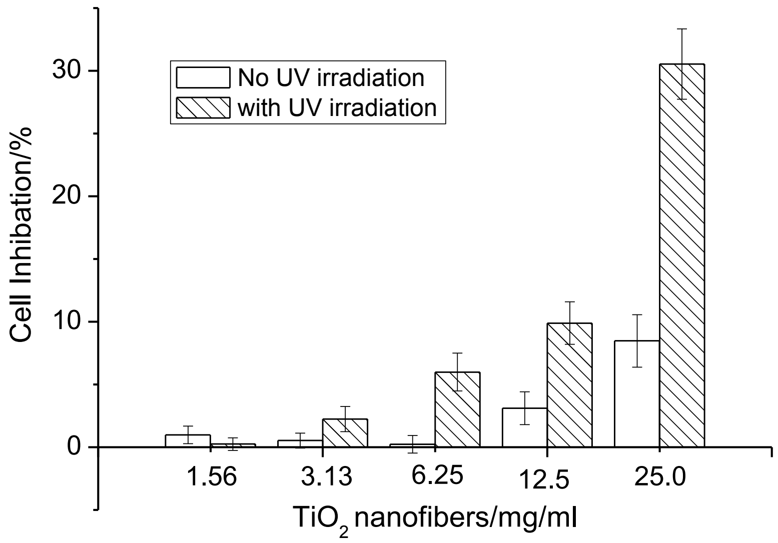

Supplement: Supplementary file 1 [file materials-07-06865-s001.pdf]
